# Effects of environmental colours in virtual reality: Physiological arousal affected by lightness and hue

**DOI:** 10.1098/rsos.230432

**Published:** 2023-10-11

**Authors:** Marieke Lieve Weijs, Domicele Jonauskaite, Ricarda Reutimann, Christine Mohr, Bigna Lenggenhager

**Affiliations:** ^1^ Department of Psychology, University of Zurich, Zurich 8006, Switzerland; ^2^ Department of Health Sciences and Technology, ETH Zurich, Zurich 8050, Switzerland; ^3^ Institute of Psychology, University of Lausanne, Lausanne 1015, Switzerland; ^4^ Faculty of Psychology, University of Vienna, Vienna 1010, Austria; ^5^ Department of Psychology, University of Konstanz, Konstanz 78457, Germany

**Keywords:** virtual reality, colour, affect, emotion, arousal, physiological measures

## Abstract

It is a popular belief that colours impact one's psychological and affective functioning. However, clear-cut scientific evidence is still lacking, largely due to methodological challenges. Virtual reality (VR) enabled us to control and modify the environment. We exposed 60 participants to red or blue environments varying in lightness and saturation. We assessed participants' physiological responses (i.e. arousal) with heart rate and skin conductance measures, and their self-reported levels of valence and arousal in response to the coloured environments. The results revealed physiological effects of lightness and hue. When compared with the baseline measures, heart rate increased, and heart rate variability decreased more in the dark than the medium lightness rooms. Both measures signalled higher arousal in the darker room, irrespective of hue. Also, when compared with the baseline measures, skin conductance increased more in the red than the blue rooms, again signalling higher arousal in the red condition. The difference between the red and the blue conditions was detectable only on some saturation and lightness combinations. We conclude that being immersed in environments of different colours can change physiological arousal. However, not all changes are driven by hue and not all the effects are measurable on all physiological parameters.

## 1. Introduction

Colours carry affective meanings on a conceptual level (e.g. [1–3]) and it is a popular belief that interior colours also impact our psychological and affective functioning. Popular psychology articles repeatedly make very general and rather bold claims. For example, on six web pages alone, we found the following statement, word-for-word: ‘Colour can have a powerful effect on the way we feel when we walk into a room. Certain shades can trigger feelings of warmth and comfort, inspire joyful spirits, or establish a soothing ambience’ [4]. Then, various media sources advise on how to choose the perfect colour for diverse purposes or claim that different colours affect our mood through physiological changes. The colour red supposedly ‘raises blood pressure, heart rate, and respiration’, while blue has the opposite effect—it ‘slow[s] your heart rate and respiration as well as lower[s] blood pressure’ [5]. The scientific evidence for colour effects on human emotions and physiology is much scarcer and the results are less straightforward than the popular psychology articles would suggest (see similar concerns expressed in [6–9]). To empirically assess the widely endorsed assumptions that colours in one's environment can impact affective states, one must use methods that can capture causal colour effects. However, there are various challenges for such studies.

The first challenge is a correct manipulation and control of the colour parameters. Colour perception is complex. People with standard colour vision perceive colours within a three-dimensional perceptual space, consisting of hue, lightness (or brightness), and chroma (or saturation; [10]). *Hue* is what people usually refer to as colour using colour terms like *red*, *green* or *blue. Lightness* describes how light or dark a colour is, while *brightness* describes how bright or dim a colour is [11]. *Chroma* defines how pure or greyish a colour is, while *saturation* is defined as the degree of colour purity relative to its lightness—chroma divided by lightness^1^. When talking about all the three colour dimensions together, or when hues cannot be disentangled from lightness and chroma, we refer to such stimuli simply as *colour*. The three perceptual colour dimensions interact with each other such that some hues exist only in certain lightness and chroma levels. For example, there are many lighter and more chromatic yellow hues than blue hues [10,12,13]. Thus, when assessing the psychological or affective impact of colour, it is always important that the colour dimensions are correctly manipulated and reported (e.g. [6,7,9,14,15]).

The second challenge is the measurement of affective states and assessment of their relationships with colour. A large body of literature showed that colours are associated with affective states, or emotions more specifically [1–3,6,14,16–27]. Such studies indicated that lighter colours were consistently associated with more positive emotions and darker colours with more negative emotions. In addition, more saturated colours were associated with more positive emotions. However, these studies measured associations between affective concepts (words) and colours (patches or terms), and thus, not with felt affective states. Some other studies included felt affective states in their design and linked those to colours [28,29]. For instance, participants had to choose the most appropriate colours for the felt affective state, which was manipulated through mood induction, and did so in a systematic way [28]. However, researchers in these latter studies also tested colour–affect correspondences and did not assess the actual impact of seen colours on felt affective states.

There is no *a priori* reason to assume that conceptual colour–affect associations equate to changes in current affective states. If we want to learn about current affective states, we need to either ask about them directly, or include physiological measures which capture changes in affective states. When asking directly, participants were often presented with a list of adjectives (e.g. happy, loving, calm, energetic, tired, interested, angry, etc.) and had to rate the extent to which each of the adjectives applied to their felt affective state (e.g. [30–32]). However, it is often difficult to categorize such affective adjectives as emotions, moods or something else. In other studies, participants directly rated their felt affective states on affective dimensions, sometimes called semantic differentials (e.g. positive–negative, calming–arousing; [33,34]). Yet in other studies (e.g. [7,35]), participants reported on their felt affective states with the self-assessment manikins (SAM; [36]). Here, participants are presented with cartoon-like figures displaying different degrees of valence, arousal and dominance. For valence, SAM figures vary from sad (i.e. negative) to smiling facial expressions (i.e. positive). For arousal, a dot in the centre of the SAM figure's chest increases in size to indicate a higher degree of excitement, while for dominance, the SAM figure itself becomes bigger and bigger to indicate a higher degree of dominance.

Alternatively to asking for self-reported current affective states, researchers used physiological measurements (table 1 for an overview and [9] for a review). Physiological measures allow for a sensitive tracking of changes in the autonomic arousal state and making inferences about felt affective states (mainly felt arousal; [49,50]). Heart rate dynamics can depict the interaction of sympathetic and parasympathetic activation, whereas skin conductance responses mainly reflect changes in arousal through sympathetic activation [51–53]. These measures have been previously used in diverse emotional contexts (e.g. [7,51,54,55]). Studies on colour effects on physiological measures produced highly diverse results. While some found red hues to be more arousing than blue hues, others did not. Likewise, some studies found lightness and saturation to be more important than hue while others did not observe any changes in physiological measures as a function of colour (table 1 for references and details).

**Table 1.** Previous studies testing the impact of perceived colours on felt affective states, using physiological recordings.

Ref	authors	year	hue	other colour parameters	colour model	testing environment	physiological recordings	length of physiological recordings	baseline	design	sample size	age (in years)	country of the study	relevant results
[37]	Wilson	1966	red, green	not controlled	unknown	projected slides on the screen	EDA	1 min for each slide (EDA recording separated into five intervals of 12 s, starting 12 s after the stimulus onset)	stimulus onset	within-subjects (two sequences of order)	20 pps (10 women)	range = 18–25	New Zealand	main effect of hue on EDA (red > green)
[38]	Jacobs & Hustmyer Jr	1974	red, yellow, green, blue	not controlled	MCS	projected slides on a white wall	ECG, EDA, respiration rate	1 min for each colour condition (ECG—30 s after stimulus onset; EDA—15 s after onset)	10 min prior to the testing session	within-subjects (four sequencies of order)	24 pps (no women)	range = 17–27	USA	main effect of hue on EDA (red > green > yellow > blue); no effect of hue on ECG or respiration
[39]	Caldwell & Jones	1985	blue, red	equalized brightness based on human perceptual judgements	Roscoe	coloured illumination on a white wall	ECG, EDA, EEG, respiration rate, finger pulse volume, eye blink frequency	45 s for each colour condition (ECG—30 s; EDA measured at four time points and averaged)	5 min prior to the testing session	within-subjects (two sequencies of order)	60 pps (30 women)	*M* = 19.5	USA	no effects of hue on ECG or EDA
[40]	Mikellides	1990	red, blue	not controlled	NCS	a physical room completely painted in colour (walls, ceilings, floors, and fitting)	ECG, EDA, EEG	20 min in each condition (analysed means)	unknown	within-subjects	24 pps (gender composition not reported)	not reported	Sweden	no effects of hue on ECG or EDA
[31]	Küller *et al.* (Study 2)	2009	red, blue	not controlled	NCS	simulated office spaces with painted walls	ECG, EEG	5 min twice (2.5 h spent in total in each room)	unknown	within-subjects on different days (order counterbalanced)	25 pps (17 women)	*M* = 23.7	Sweden and UK	main effect of hue on ECG (blue > red)
[41]	AL-Ayash *et al*.	2016	red, yellow, blue + white	each hue appearing as vivid and pale	NCS	coloured physical panels in a testing space (cubicle)	ECG	heart rate measured for an unknown duration after 5 min of exposure	5 min prior to the testing session	within-subjects on different days (order counterbalanced)	24 pps (13 women)	range = 20–38	Australia	main effect of hue on ECG (red = yellow > blue); no differences between vivid and pale conditions
[42]	Zieliński	2016	red, green, blue, yellow	for each hue, two levels of lightness and two levels of chroma	NCS	colours presented on screen, filling the entire space	EDA	10 s for each colour condition, averaged across the three presentations of each colour condition.	5 min prior to the testing session	within-subjects, each colour condition seen three times	64 pps (all women)	*M =* 22.5	Poland	no main effect of hue on EDA; main effect of chroma on EDA (more chromatic > less chromatic)
[7]	Wilms & Oberfeld	2018	red, blue, green + grey	each hue, three levels of brightness, three levels of saturation	CIE *Lab*	laboratory testing spaces (cubicles), colours manipulated by lights	ECG, EDA	30 s for each colour condition (ECG—6 s after stimulus onset)	6 s before the stimulus onset	within-subjects (order counterbalanced)	62 pps (42 women)	*M* = 23.4	Germany	no effect of hue on ECG; main effects of saturation and brightness on ECG (high > medium > low, for both parameters); no effect of hue on EDA
[43]	Cha *et al*.	2020	red, green, blue + white	not controlled	NCS	simulated rooms in VR	ECG	2 min for each colour condition	5 min prior to and in between the testing sessions	within-subjects (order counterbalanced)	55 pps (7 women)	*M* = 21.3	Hong Kong, China	no effect of hue on ECG
[44]	Llinares *et al*.	2021	four warm and four cold hues	For each hue, two levels of chroma; matched in lightness	MCS	simulated rooms in VR	ECG	1.5 min for each colour condition	3 min prior to the testing session	between-subjects; each participant saw three rooms	160 pps (67 women); at least 20 pps per colour condition	*M* = 23.6	Spain	main effect of cold/warm colours on ECG (cold > warm)
[45]	Oh *et al*.	2021	red, yellow	for each hue, two levels of brightness and three levels of chroma	MCS	laboratory testing spaces (cubicles), colours manipulated by paint	ECG	2.5 min for each colour condition	prior to the testing session (unreported duration)	within-subjects (fixed order)	27 pps (13 women)	*M =* 22.0	Korea	no effect of colour on ECG
[46]	Bower *et al*.	2022	blue + white	not controlled	commercial colours	simulated rooms in VR	ECG, EDA, respiration rate	2 min for each condition	prior to the testing session (unreported duration)	within-subjects (order counterbalanced)	18 pps (8 woman)	*M =* 34.5	Australia	main effect of colour on ECG, EDA and respiration (blue > white)
[47]	Jiang *et al*.	2022	21 colours: shades of red, yellow, blue, green, purple, brown + grey	not controlled	commercial colours	simulated rooms in VR	ECG	3 min for each colour condition	3 min prior to the testing session	within-subjects (order counterbalanced)	70 pps (29 women)	range = 22–27	China	main effect of colour condition on ECG (red, purple, yellow more arousing; grey and brown less arousing)
[48]	Oh & Park	2022	green, blue	for each hue, two levels of brightness and three levels of chroma	MCS	laboratory testing spaces (cubicles), colours manipulated by paint	ECG	2.5 min for each colour condition	prior to the testing session (unreported duration)	within-subjects (fixed order)	24 pps (15 women)	*M =* 21.4	Korea	effect of chroma, only in the blue condition (higher arousal with higher chroma)

*Note*. pps = participants; *M* = mean; ECG = electrocardiogram (heart activity); EDA = electrodermal activity (skin conductance); EEG = electroencephalogram (neural activity); CIE = Commission international de l'éclairage; NCS = natural colour system, MCS = Munsell colour system. A plus symbol (+) is used to separate hues (chromatic colours) from achromatic colours (i.e. white, grey, black).

Finally, it is also not an easy task to experimentally immerse participants in coloured spaces, and this can be achieved either by changing colours in physical rooms [7,30–32,35,37,40,41,45,48] or by simulating coloured environments in virtual reality (VR; [32,33,43,44,46,47]). When it comes to physical spaces, one must be able to change interior colours of the same room in a consistent, reproducible and timely manner. One should also control for the weather conditions outside and the time of the year, as this would change the quality of light entering the room (see an example in [56]). Relevant to physical and virtual environments, one should control for the indoor temperature, the duration of time spent in each coloured environment, and participants' activities while in the environment. Furthermore, although tempting, one should not change any other element of the room apart from the colours, because one would not know what caused (if any) psychological or affective changes under different colour conditions.

Overall, running such well-controlled studies is demanding and it requires high investment in terms of money and time. These numerous methodological challenges probably explain why we found few well-controlled empirical studies that could inform us on affective changes in response to colour [7,9,30–33,35,37–48]. Among these, even fewer studies used physiological measurements and experimentally manipulated and analysed lightness and/or saturation in addition to hue ([7,41,42]; table 1). And from the latter, none implemented the VR technology. VR would be a powerful tool to overcome these difficulties as it enables high experimental control and high ecological validity. Overall, there was a high heterogeneity in testing environments, exposure duration, tested colours and outcomes (table 1).

In the current study, we exposed participants to coloured rooms in the VR environment and collected physiological and self-report correlates of felt affective states. We immersed participants for 6 min in either red or blue VR rooms, and each hue appeared at two lightness (medium, low) and two saturation (high, medium) levels. Each participant saw only one hue (i.e. red or blue) to make the study aim less obvious. We were interested in the potentially arousing effects of red, as reported in some previous studies (table 1), and wanted to contrast this hue with another hue. We chose blue because it is often assumed to have calming effects. To increase comparability with previous studies, we implemented the same colours as in Wilms and Oberfeld's study [7], albeit a reduced set of them. While participants were immersed in the VR rooms, we continuously collected their physiological reactions—heart rate (HR), heart rate variability (HRV) and skin conductance—for 3 min. After the 3 min, participants each time provided self-reported SAM ratings for arousal and valence [36], as these dimensions are the most pertinent to felt affective states [57,58]. They also rated their felt presence (similar to [59]).

Considering the high degree of heterogeneity of previous findings (table 1), we could not formulate clear-cut hypotheses. Based on the literature on conceptual colour–emotion associations (e.g. [1,6,16]), we nevertheless expected red hues to be more arousing than blue hues (also see, [60]). We would also expect darker and more saturated colours to be more arousing than lighter and less saturated colours. More precisely, for the conditions of higher arousal we expected higher HR and lower HRV responses as well as enhanced electrodermal activity (EDA).

## 2. Methods

### 2.1. Participants

We retained a final sample of 60 participants (39 female) with a mean age of 27.6 years (s.d. = 10.7 years). We had recruited four additional participants (four men) but excluded them due to (i) technical issues (*n* = 1), (ii) lack of understanding the instructions (*n* = 1), and (iii) impaired colour vision (*n* = 2), as tested with the Ishihara test for colour vision deficiency [61]. The 60 participants had normal or corrected-to-normal vision, and no current or previous history of psychiatric or neurological disease. We recruited this typical convenience sample from the Swiss population, which consisted of 33 students and 27 people employed in diverse occupations. Student participants received course credits in exchange for their participation. The experimental protocol was approved by the Ethics Committee of the Faculty of Arts and Social Sciences at the University of Zurich (no. 17.12.15), and participants provided informed consent prior to their participation. Data were collected from June to September 2020. Due to the COVID-19 pandemic, we were restricted on how many participants could be collected in this timeframe. Nevertheless, the sample size was similar to or larger than those in the previous related studies (table 1; [7]).

### 2.2. Design

We used a mixed factor 2 × 2 × 2 design. To keep the experiment at a reasonable length, we had to consider both within-subject and between-subject factors. Importantly, we wanted to minimize the ease at which participants could guess the major goal of the experiment (i.e. psychological and physiological responses to rooms of different colours). Since hue changes are more evident than changes in lightness or saturation, the within-participants factors were lightness (low and medium) and saturation (medium and high), and the between-participants factor was hue (blue or red). Thirty-one participants were in the red condition, and 29 in the blue condition. To avoid order effects, for each hue, we presented the four within-participant conditions in four different predefined orders (see electronic supplementary material, table S1).

### 2.3. Set-up and apparatus

The experimental session took place in a testing room at the university. Participants were sitting on a chair in a quiet room with a mean indoor temperature of 22.1°C (s.d. = 1.3°C). For the VR stimulation, we used a head-mounted display (HMD) Oculus Rift CV1. Participants used one Oculus controller in the dominant hand. The experimental stimuli were created with Unity (v. 2018.2.8f1). The VR simulation was run on an Alienware 15 R3 computer (Nvidia Geforce GTX 1080 8GB; 16GB RAM; Intel Core i7; Windows 10).

### 2.4. Colour stimuli

**Figure 1.**
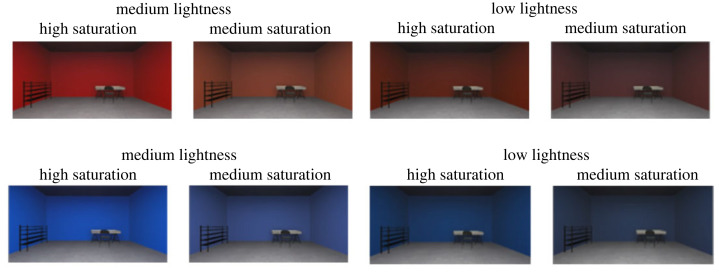
Virtual colour rooms, varying in hue, saturation, and lightness, in which participants were immersed. Half of the participants explored all four red rooms while the other half explored all four blue rooms. Rooms were presented in counterbalanced order and thus the order differed for each participant (see also figure 2).

We created a virtual environment consisting of a room (6 × 6 × 3.3 ratio) in which colour had been applied to all vertical walls. We designed a grey mesh colour resembling concrete for the floor and ceiling (figure 1). We selected colours previously used in a related study with physiological measures [7]. For the red and blue hue conditions, we created four room conditions by manipulating saturation (medium and high) and lightness (low and medium). To avoid uncomfortable strain to the eyes, we did not use hues at the highest lightness levels used in this previous study [7]. To render these colours in VR, we converted the colours reported in the CIE *LCh* colour space into the sRGB colour space with an online conversion tool, assuming standard viewing conditions ([62]; table 2). We simulated the light sources in the virtual room to resemble a real room and kept the light conditions constant across the experimental conditions. When in the VR environment, participants could move their heads, meaning that colours slightly varied with these movements due to applied rendering to create shadows.

**Table 2.** Colorimetric values of the colour stimuli, rendered in the VR environment.

hue	lightness level	saturation level	colorimetric values (CIE *LCh* colour system)		
			L*	sat (C*/L*)	h* (in degrees)
blue	medium	high	34.99	2.28	290.49
blue	medium	medium	34.99	1.39	291.64
blue	low	high	19.96	2.34	291.06
blue	low	medium	19.89	1.45	285.91
red	medium	high	34.98	2.35	44.69
red	medium	medium	35.02	1.43	42.40
red	low	high	20.01	2.42	42.97
red	low	medium	19.94	1.31	23.49

*Note.* The table presents colorimetric values as used by Wilms and Oberfeld [7] according to the CIE 1976 *LCh* system. These values were transformed to the sRGB space, which were applied in the VR environment.

### 2.5. Measures

#### 2.5.1. Self-report measures

We used the self-assessment manikin (SAM) to assess subjective levels of valence and arousal [36]. The non-verbal, pictorial SAM ratings consist of nine cartoon-like pictures which vary from a sad to a smiling face for valence, and from a figure with a small dot in the chest area to a figure with an explosion in the same area for arousal. Participants were asked to select one of the nine figures they find most appropriate for their level of felt valence and arousal. Their choices were later converted to values from 1 (lowest level of valence or arousal) to 9 (highest level of valence or arousal). We were interested in the change in arousal and valence from the baseline, when exposed to different VR rooms. Thus, we subtracted the baseline rating from the rating in the experimental conditions, so that higher values indicate an increase in arousal and valence as compared with the baseline.

#### 2.5.2. Physiological measures

We recorded the electrocardiogram (ECG) and electrodermal activity (EDA) concurrently with a Biopac MP150 System with EDA100C and ECG100C amplifiers (Goleta, USA). Signals were recorded with a sampling rate of 500 Hz in the Acqknowledge software (v. 4.1, Goleta, USA). For the EDA recording, we placed two Ag/AgCl electrodes with isotonic paste on the palmar surfaces of the distal phalanges of the first and second digit of the non-dominant hand. ECG was recorded with three disposable Ag/AgCl electrodes, placed on the left and right clavicle and lowest left rib. To mark the beginning of a new experimental condition, we sent triggers from the laptop running the experiment to the recording software using an MMB Triggerbox (Neurospec, Stans, Switzerland).

The ECG data were pre-processed with the MATLAB extension HRVTool [63]. We verified automatic R-peak detection using visual inspection. We used the 3 min baseline segment, and the 3 min room exploration segments for subsequent analysis. For each segment, we calculated heart rate (HR) and the root mean square of the successive differences (RMSSD) as an index of heart rate variability (HRV). For both HR and HRV, change was defined as the difference between baseline and experimental condition, with higher values indicating an increase in the respective measure as compared with baseline.

From the EDA recording, we calculated the sum of the amplitude of skin conductance responses (SCR) within the 3 min baseline and room exploration segments. EDA data were pre-processed using the MATLAB extension Ledalab [64]. They were filtered with a first-order Butterworth low-pass filter with a lower cut-off frequency of 5 Hz. Manual artefact correction was applied if necessary to smoothen electric field artefacts. We used a through to peak analysis [65], the sum of the amplitude of SCRs greater than 0.05 µS to obtain the electrodermal activity within the 3 min baseline segment, and 3 min room exploration segments. EDA change was defined as the difference between baseline and experimental condition, with higher levels indicating an increase in EDA as compared with baseline.

### 2.6. Procedure

Participants were tested in a quiet, well-aired 26 m^2^ room, with an average indoor temperature of 22.1°C. They stayed seated during the entire experiment but could freely move their heads. Due to the COVID-19 pandemic, participants and the experimenter wore surgical masks during the entire experiment. They maintained at least 2 m distance, apart from when electrodes were applied or taken off. All non-reusable materials were disinfected before use and participants had been screened for COVID-19.

**Figure 2.**
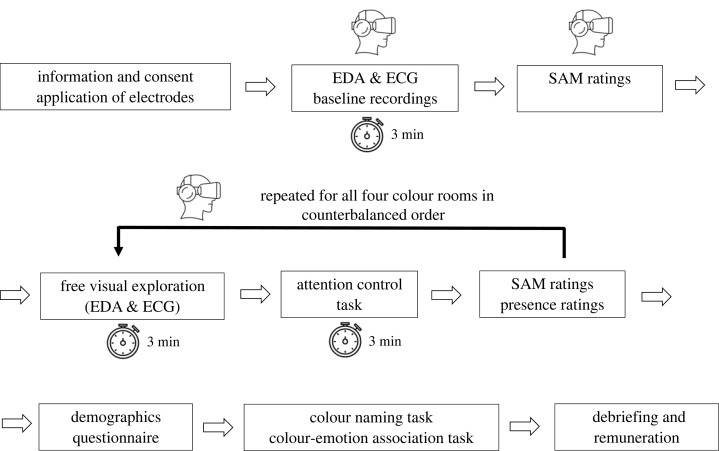
Procedure of the experiment. VR goggle icon marks tasks which participants completed in VR. See virtual rooms in figure 1. EDA = electrodermal activity; ECG = electrocardiogram; SAM = self-assessment manikins [36].

Upon the start of the experiment, participants received general study information that did not mention colours (figure 2). Then, they provided written informed consent and proceeded to the experiment, which was always conducted by the same experimenter (R.R.). As the first step, the experimenter applied all electrodes to record physiological signals before putting on the HMD, which was used for presenting the virtual environment. Participants used either a hand-held controller or the head-tracking of the HMD to give responses. We displayed the instructions in the virtual setting on a white panel in front of the black background.

Once within the virtual environment, we started by recording the baseline measures (figure 2). First, we recorded ECG and EDA while participants looked at the black screen in the HMD. We instructed them to stay still during the 3 min of recording. Then, they completed the SAM measures and the bodily maps of emotion [66]. Unfortunately, we could not further analyse the latter because of the missing data in 28 participants due to technical difficulties.

After the baseline measures, we immersed participants into the first of the four coloured rooms (figure 2). Hue depended on the condition, while lightness and saturation were presented in counter-balanced order (figure 1). Once seated, they were instructed to slowly move their head and freely explore the room for 3 min. This duration is in line with recent studies in the field ([41,43–48]; table 1). Subsequently, they received instructions on the sustained attention task [67], which they performed for another 3 min, to ensure they were focused at all times. After the 6 min of exposure, participants completed the SAM and the bodily maps of emotion. They also reported how *present* they felt in the virtual room on a visual analogue scale ranging from 0 to 100 (similar to [59]). Upon completion of these measures, participants were immersed into the next coloured room, and we repeated the procedure in the same order.

After having been exposed to all four coloured rooms, participants removed the HMD and responded to additional questions on the computer screen (figure 2). We collected demographic data and checked if participants were aware of the purpose of this experiment. To ensure that participants recognized the colours correctly, we added a colour naming task of the previously seen colours, presented as rectangular patches (see electronic supplementary material, table S8). Furthermore, they rated the degree to which they associated each colour patch with *happiness*, *anger*, *sadness*, *fear*, *surprise* and *disgust* (the basic emotions proposed by [68]), using a five-point Likert scale. These ratings are reported in electronic supplementary material, figure S1. At the end of the experiment, participants were thanked, debriefed and received their compensation. The experiment took approximately 60 min to complete.

### 2.7. Data analyses

#### 2.7.1. Data cleaning

Due to measurement errors, we did not have complete data for all measures of all 60 participants. Thus, we had a varying number of data points for the analyses, which we accounted for by using linear mixed models for the statistical analyses. For the SAM measure, 59 participants were included, one had to be excluded due to a missing response in the baseline measure. In the EDA analyses, 46 participants were included, we had to exclude two participants due to missing triggers, and 12 due to an error in the recording. In the ECG analyses, 57 participants were included, two participants had to be excluded due to missing triggers and one due to an error in the recording.

#### 2.7.2. Cover story check

When asked about the purpose of the experiment, 17 participants assumed that changing colour was part of the research question. Eight of them further related colour changes to emotions. Nevertheless, we analysed all the data to maintain sufficient power for the statistical analyses.

#### 2.7.3. Statistical analyses

We ran the following linear mixed models for the different outcome measures—SAM valence, SAM arousal, felt presence, EDA, HR and HRV:

y∼Hue×Saturation×Lightness +Order+(1|ID).

More specifically, we included three factors as predictors: hue (two between-subject levels: red and blue), saturation (two within-subject levels: high and medium) and lightness (two within-subject levels: medium and low), as well as their two-way and three-way interactions. To control for the presentation order, we added another factor—order (four within-subject levels; see electronic supplementary material, table S1). We completed the model with the random intercept for participants to account for the repeated-measures design. We followed up with *post hoc* tests using Tukey correction. To compare changes in experimental conditions from baseline, we ran one-sample *t*-tests where appropriate and reported estimated marginal means (EMM).

#### 2.7.4. Transparency and openness

We report how we determined our sample size, all data exclusions, all manipulations and all measures in the study. All data, analysis code and research materials are available at https://osf.io/k5xqz [69]. Statistical analyses were performed in R v. 4.0.2 [70], and we used the lmerTest package [71] for the linear mixed model analyses. The alpha level was set to 0.05, and *p*-values were calculated using Satterthwaite's approximation. The study design and its analysis were not pre-registered.

## 3. Results

### 3.1. Participants

We compared basic demographic information between participants recruited in the red and the blue conditions (between-subjects). The two groups of participants did not differ in age, *t*_58_ = 0.222, *p* = 0.825, gender composition, $\chi_{1}^{2}=0.007,$ *p* = 0.935, or whether they were students, $\chi_{1}^{2}=1.13,$ *p* = 0.287.

### 3.2. Self-report measures

#### 3.2.1. Self-assessment manikin arousal

The linear mixed model assessing the effects of hue, saturation, and lightness on change in arousal from baseline did not reveal any significant effects of these variables or their two-way or three-way interactions. There was, however, a significant main effect of order, *F*_1,173_ = 17.06, *p* < 0.001, demonstrating that self-reported arousal was the highest at the first presentation, then gradually diminishing towards the baseline level, *b* = −0.27, 95% CI = [−0.40 −0.14] (see the full model in electronic supplementary material, table S2; table 3 for descriptive statistics of SAM arousal per condition).

**Table 3.** Descriptive statistics of self-reported arousal, valence and presence.

hue	lightness level	saturation level	SAM arousal		SAM valence		presence	
			*M*	95% CI	*M*	95% CI	*M*	95% CI
blue	medium	high	3.48	2.83–4.13	5.66	5.23–6.08	49.3	39.1–59.5
blue	medium	medium	3.59	2.86–4.32	6.10	5.66–6.55	51.7	41.7–61.6
blue	low	high	3.72	2.95–4.50	5.90	5.40–6.39	53.0	43.6–62.4
blue	low	medium	3.76	3.04–4.47	5.93	5.38–6.48	49.6	39.9–59.3
red	medium	high	3.55	2.90–4.20	6.03	5.62–6.44	51.3	42.2–60.4
red	medium	medium	3.39	2.78–4.00	6.00	5.58–6.42	48.4	38.4–58.3
red	low	high	3.65	3.05–4.24	6.16	5.75–6.58	51.3	42.5–60.1
red	low	medium	3.74	3.15–4.33	6.19	5.80–6.59	51.4	42.7–60.1

#### 3.2.2. Self-assessment manikin valence

In analogy to the SAM arousal ratings, the linear mixed model assessing the effects of hue, saturation and lightness on change in valence from baseline did not reveal any significant effects of these variables or their two-way or three-way interactions. Again, there was a significant main effect of order, *F*_1,173_ = 68.80, *p* < 0.001, demonstrating that self-reported valence was the highest at the first presentation, then gradually diminishing towards the baseline level, *b* = −0.33, 95% CI = [−0.41 −0.25] (see the full model in electronic supplementary material, table S3; table 3 for descriptive statistics of SAM valence per condition).

#### 3.2.3. Presence

The linear mixed model assessing effects of hue, saturation and lightness, showed no significant main and no interaction effects on self-reported presence in the virtual room (all *p*s > 0.11, see electronic supplementary material, table S4 for the full model). Overall, presence was at an intermediate level (table 3).

### 3.3. Physiological measures

#### 3.3.1. Heart rate and heart rate variability—electrocardiogram parameters

**Figure 3.**
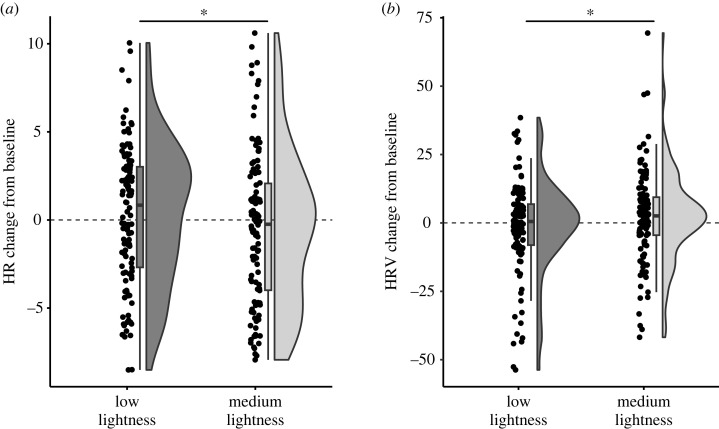
(*a*) Heart rate (HR) and (*b*) heart rate variability (HRV) changes, compared with the baseline, across the lightness conditions. Differences between the conditions were significant at *p* < 0.050 (marked with *). HRV was assessed as RMSSD.

The linear mixed model showed a significant main effect of lightness on HR, *F*_1,164_ = 6.35, *p* = 0.013. The HR level, relative to the baseline, was higher in the low lightness condition, EMM = 0.24, 95% CI = [−0.77 1.26], compared with the medium lightness condition, EMM = −0.49, 95% CI = [−1.50 0.53]; (figure 3). However, the HR did not significantly differ from the baseline, either in the low lightness condition, *t*_113_ = 0.71, *p* = 0.48, or in the medium lightness condition, *t*_113_ = −1.30, *p* = 0.20. Additionally, there was a significant main effect of order on HR, *F*_1,164_ = 4.89, *p* = 0.029, with HR decreasing across repetitions, *b* = −0.30, 95% CI = [−0.55 −0.06]. The main effects of hue and saturation on HR were not significant and neither were the two-way and three-way interactions (see electronic supplementary material, table S5 for all model coefficients).

The linear mixed model showed a significant main effect of lightness on HRV (measured as RMSSD), *F*_1,164_ = 5.97, *p* = 0.016. The HRV level was lower in the low lightness condition, EMM = −1.20, 95% CI = [−5.20 2.79], than in the medium lightness conditions, EMM = 2.10, 95% CI = [−1.89 6.09] (figure 3). However, the HRV did not significantly differ from the baseline in either the low lightness condition, *t*_113_ = −0.79, *p* = 0.43 or in the medium lightness condition, *t*_113_ = 1.44, *p* = 0.15. All other main effects as well as the two-way and the three-way interactions were not significant (see electronic supplementary material, table S6 for all model coefficients).

#### 3.3.2. Skin conductance—electrodermal activity

The linear mixed model showed a significant main effect of hue on EDA, *F*_1,46_ = 4.41, *p* = 0.041. This effect demonstrated that EDA was higher in the red, EMM = 3.45, 95% CI = [1.92 4.97], than blue conditions, EMM = 1.29, 95% CI = [1.92 4.97].

**Figure 4.**
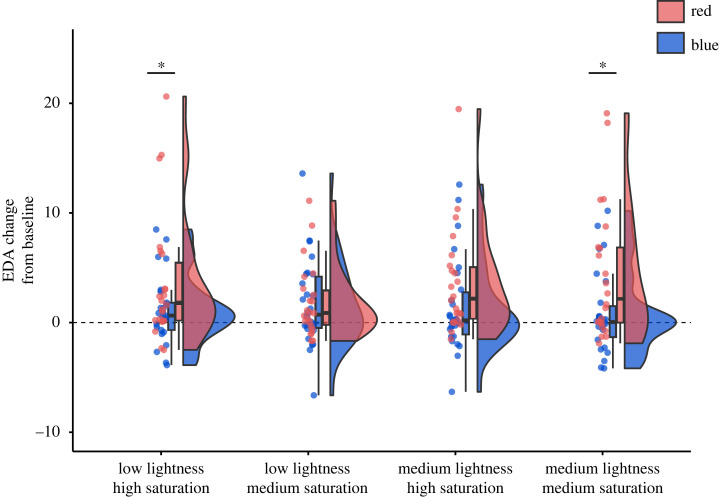
Skin conductance changes across the colour conditions (hue, saturation and lightness). Differences between some conditions were significant at *p* < 0.050 (marked with *).

There was also a significant three-way interaction of hue, saturation and lightness, *F*_1,136.21_ = 4.04, *p* = 0.046; figure 4. *Post hoc* tests showed that the higher EDA response in the red as compared with the blue conditions was present only in the low lightness and high saturation, *t* = −2.69, *p* = 0.044, and the medium lightness and medium saturation, *t* = −2.69, *p* = 0.008, conditions. In the medium lightness and high saturation condition, the effect was in the same direction, but it was not significant, *t* = −1.67, *p* = 0.098. In the low lightness and medium saturation condition, there was no significant difference between the red and the blue conditions, *t =* −0.17, *p =* 0.87. When compared with the baseline, the EDA response was significantly higher in all four red hue conditions (*p*s ≤ 0.026, adjusted for multiple comparisons) while it was not significantly higher in any of the blue hue conditions (*p*s ≥ 0.064, adjusted for multiple comparisons). The other main effects and the two-way interactions were not significant (see electronic supplementary material, table S7 for model coefficients).

### 3.4. Correlations between the self-reported and physiological measures

We used Spearman method to calculate correlations between the self-report measures (SAM valence and arousal) and the physiological indices (HR, HRV and EDA). We found a significant positive correlation between SAM arousal and HR, *r* = 0.14, *p* = 0.021. All other correlations were not significant (all *p*s > 0.31; table 4).

**Table 4.** A correlation table with Spearman correlation coefficients (*r*) of self-report and physiological measures of change in felt emotion.

	HR	HRV (RMSSD)	EDA
SAM arousal	0.14*	−0.03	−0.07
SAM valence	−0.03	0.02	0.03

*Note*: *p ≤ 0.050.

## 4. Discussion

Colours are presumed to influence affective states. However, the scientific evidence for such causal effects is sparse (e.g. [9,15,35,72–74]). This might be due to methodological challenges, related to both colour manipulation and a reliable measurement of affective states. A solid design would imply that colours of the same environmental space could be manipulated in a consistent manner, ideally accounting for the individual contributions of hue, saturation and lightness (e.g. [6–8]). To this end, we manipulated wall colours in a VR setting, immersing participants in either red or blue rooms, varying in degrees of lightness and saturation. To assess participants' affective states, we recorded their physiological arousal with heart rate (HR and HRV) and skin conductance (EDA) measures. We also asked them to report how they felt both in terms of valence and arousal, as well as how present they felt in each virtual room.

We found differences as a function of room colour for physiological measures (HR, HRV and EDA), but not for subjective self-report measures (valence, arousal and presence). Our major results consisted of two significant effects, one of lightness on heart rate measures and another one of hue on skin conductance measures. In both cases, we determined the physiological variables as a change from baseline (3 min rest before the experiment). For the first effect, the heart rate was higher, and the heart rate variability was lower in the dark rooms as compared with rooms of medium lightness. These differences indicated that participants experienced higher arousal in the dark than medium light rooms, irrespective of whether the rooms were red or blue. For the second effect, the skin conductance responses were higher in the red as compared with the blue rooms. This difference between the two hue conditions was further specified by an interaction of hue with lightness and saturation. Participants’ skin conductance increased more in red rooms of low lightness and high saturation as well as red rooms of medium lightness and medium saturation, as compared with the blue rooms with the same lightness and saturation combinations. These results signified higher arousal in the red than blue rooms. We tested whether this observation could be further confirmed by comparing the physiological response in the experimental conditions with the one at baseline. And indeed, they showed increased skin conductance responses in the red rooms, but not in the blue rooms, signalling a heightened state of arousal in the red rooms.

In table 1, we depicted key results from previous studies assessing psychophysiological responses in different coloured environments (also see an early review in [9]). These previous studies had used heterogeneous experimental designs, reaching heterogeneous results. Accordingly, we were hesitant to use these studies to formulate *a priori* hypotheses. Instead, we considered the literature on conceptual colour–emotion associations (e.g. [6,16]). We predicted (i) red hues to be more arousing than blue hues, (ii) darker colours to be more arousing than lighter colours, and (iii) more saturated colours to be more arousing than less saturated colours. Our results on the skin conductance measure confirmed the first prediction that red rooms were more arousing than blue rooms. Our results on the heart rate measures confirmed the second prediction that darker rooms were more arousing than lighter rooms. We did not confirm the third prediction that more saturated rooms would be more arousing than less saturated rooms. The first two effects were observed for only one of the two physiological measures (not both), yet such selective confirmation is not uncommon (table 1, and also [49,75,76]). This is because affective states are characterized by highly specific and dissociable response patterns across indices of autonomic nervous system activity (see [49] for examples).

Previous studies on the impact of colour on physiological responses resulted in heterogeneous findings (table 1). There is little surprise then that our results also differed from many of these former studies. To compare our results with the previous literature, we detailed two studies, which tested for hue effects in environmental spaces by also controlling for saturation and/or lightness [7,41]. For the result on lightness, we observed higher physiological arousal for darker environmental spaces, while Wilms and Oberfeld [7] reported higher arousal for brighter spaces. Our results were detected with heart rate measures and not with skin conductance measures, while Wilms and Oberfeld's [7] results were detected with skin conductance and not heart rate measures. The study by AL-Ayash *et al*. [41] was not relevant here, because they did not manipulate brightness. The inconsistencies between studies might be due to exposure duration. We had longer exposure duration, potentially rendering our darker spaces more threatening and thus more arousing [77–79]. Wilms and Oberfeld [7] immersed participants in colours for only 30 s comparing the physiological responses with the time window immediately prior. Perhaps, their increased arousal for brighter spaces reflected physiological responses to change, common in humans as well as animals when exposed to bright stimuli (e.g. startle reflex, [80–82]). Further studies manipulating the exposure time are necessary to confirm these speculations.

For the second result on hue, both the current study and AL-Ayash *et al*.'s [41] study showed red hues to be more physiologically arousing than blue hues. The study by Wilms and Oberfeld [7] did not detect a statistically significant difference between red and blue hues. Again, we detected the hue effect with skin conductance measures and not with heart rate measures, while AL-Ayash and colleagues' [41] study detected the hue effect with heart rate measures. They did not collect skin conductance measures. At this point, we cannot know the reasons behind the discrepant findings between studies, with possible causes being differences in testing environments and locations, exposure durations, colour stimuli, small sample sizes and others.

### 4.1. Limitations, challenges and future directions

Obvious limitations of the current study were the between-subjects design, testing only two hues, and the generalization of results. We tested hue as a between-subjects variable because we did not want to make it obvious that we study colours. Accordingly, participants were exposed to a single hue, but of different lightness and saturation levels. Therefore, differences between the hue conditions might have been due to this between-subject design. It is worth noting, participants in the two hue conditions did not differ on obvious demographic variables, like age, gender or student status. For hue, we could not conclude that our results were specific to red and blue hues, because we did not test other hues. Perhaps, our results were more general and could be applicable to warm and cool colours. In that case, one would expect that exposure to other warm colours (e.g. orange, yellow, pink) would increase physiological arousal as found for the red hue. Indeed, two previous studies [41,47] reported that red, yellow and purple hues were more arousing than blue hues (table 1; but cf. [44]).

Furthermore, we could not know whether our results generalize to other populations and settings. We tested German speakers in Switzerland, where white wall paint is standard [83]. Thus, results from this population may or may not apply to people coming from other social, linguistic and/or geographical environments, especially those beyond the well-studied WEIRD populations [84]. That said, we had formulated and confirmed predictions based on the results that conceptual colour–emotion associations were widely shared across 30 nations [3,20]. Thus, our current results might replicate in other populations.

The transfer of our results to applied settings (e.g. interior design) is not trivial. First, the current study manipulated wall colours in virtual reality, and it remains unknown whether the same effects would be replicated in physical environments. Then, there are other differences between laboratory studies and real environments. In laboratory studies, participants are usually exposed to environmental colours for short periods of time (table 1). In real life, people spend prolonged periods of time in the same spaces, probably becoming habituated to these colours over time. With habituation, affective impact of colours probably lessens too (e.g. affective habituation [85]; art fatigue effect [86]). And indeed, such affective habituation processes might explain why colours of student dormitories showed no influence on their affective states over a testing period of 13 months [30]. Moreover, in real life, colours have been chosen by their inhabitants (e.g. homes) or imposed by others (e.g. offices, shops, restaurants), while in laboratory studies they have nearly always been imposed by researchers. Based on the literature on situation selection [55,87], the affective impact of colours on felt affective states would probably be stronger when participants select colours themselves, especially when they find the resultant environment aesthetically pleasing [88,89].

## 5. Conclusion

The current study is promising in demonstrating that (virtual) wall colours can impact participants’ psychophysiological states, and by inference, felt emotions. Hue as well as lightness and saturation are important in this regard (also see, [7]). However, with only a handful of published studies in the field, the understanding of how, when, and why colour influences felt emotions is still in its infancy. The field calls for systematic investigations, using consistent research designs across multiple studies, employing a large array of colours manipulated on all three colour dimensions, studying diverse and sufficiently large samples of participants, and testing various environmental situations (e.g. VR, real spaces). Currently, we are unaware how long colour effects last, especially when considering the initial light and startle reflexes and affective habituation over time. Finally, we know nearly nothing about individual differences, such as the role participants' aesthetic preferences and self-choice play in affective impact of colours. In our view, much more fundamental research would be needed before results can be transferred to applied domains such as interior design or healthcare.

## Data Availability

All data have been made publicly available at the Open Science Framework and can be accessed at https://osf.io/k5xqz/. The data are provided in electronic supplementary material [90].

## Footnotes

1 Despite the subtle differences, in the scientific literature and in common language, *lightness* and *brightness* as well as *chroma* and *saturation* are used interchangeably. In this article, when describing others’ works, we used the terms originally used by the authors. When describing our current study, we used *lightness* and *saturation*.
